# Artemether, Artesunate, Arteannuin B, Echinatin, Licochalcone B and Andrographolide Effectively Inhibit SARS-CoV-2 and Related Viruses *In Vitro*

**DOI:** 10.3389/fcimb.2021.680127

**Published:** 2021-08-30

**Authors:** Yunjia Hu, Meiqin Liu, Hongbo Qin, Haofeng Lin, Xiaoping An, Zhengli Shi, Lihua Song, Xinglou Yang, Huahao Fan, Yigang Tong

**Affiliations:** ^1^Beijing Advanced Innovation Center for Soft Matter Science and Engineering, College of Life Science and Technology, Beijing University of Chemical Technology, Beijing, China; ^2^Chinese Academy of Sciences (CAS) Key Laboratory of Special Pathogens, Wuhan Institute of Virology, Center for Biosafety Mega-Science, Chinese Academy of Sciences, Wuhan, China; ^3^Savaid Medical School, University of Chinese Academy of Sciences, Beijing, China

**Keywords:** COVID-19, SARS-CoV-2, GX_P2V, traditional Chinese medicine, antivirals

## Abstract

Since the first reported case caused by the novel coronavirus SARS-CoV-2 infection in Wuhan, COVID-19 has caused serious deaths and an ongoing global pandemic, and it is still raging in more than 200 countries and regions around the world and many new variants have appeared in the process of continuous transmission. In the early stage of the epidemic prevention and control and clinical treatment, traditional Chinese medicine played a huge role in China. Here, we screened out six monomer compounds, including artemether, artesunate, arteannuin B, echinatin, licochalcone B and andrographolide, with excellent anti-SARS-CoV-2 and anti-GX_P2V activity from Anti-COVID-19 Traditional Chinese Medicine Compound Library containing 389 monomer compounds extracted from traditional Chinese medicine prescriptions “three formulas and three drugs”. Our discovery preliminary proved the stage of action of those compounds against SARS-CoV-2 and provided inspiration for further research and clinical applications.

## Introduction

Coronavirus is a type of positive-sense, single-stranded RNA virus (Woo et al., 2010) that can infect mammals and birds and cause respiratory, nervous, and digestive diseases (Weiss and Navas-Martin, 2005; Cavanagh, 2007; Fehr and Perlman, 2015). Currently, there are 7 types of coronaviruses known to infect humans, ranging from OC43, HKU1, 229E, and NL63 that produce the generally mild symptoms of the common cold (Pene et al., 2003; Abdul-Rasool and Fielding, 2010; Lau et al., 2011; Lim et al., 2016) to more aggressive SARS-CoV, MERS-CoV, and SARS-CoV-2 that cause severe respiratory symptoms (Peiris et al., 2003; Wong et al., 2019; Lai et al., 2020). Especially the recent outbreak of COVID-19 caused by SARS-CoV-2, which was first reported in December 2019 in Wuhan, has resulted in an ongoing global pandemic. As of 3 February 2021, more than 103 million cases have been reported across 216 countries and territories, resulting in 2,252,923 deaths. What is worrying is that in the process of continued transmission, mutations occurred at multiple sites on the virus genome, resulting in six variants including B.1.1.207, B.1.1.7, Cluster 5, 501.V2, P.1 and B.1.429/CAL.20C (Koyama et al., 2020; Tang et al., 2020), which may have a potential impact on ongoing drug and vaccine development (Li et al., 2020; Weisblum et al., 2020; Yurkovetskiy et al., 2020).

Traditional Chinese medicine (TCM) has a long history and is widely used in East Asia, especially in China, saving countless lives for thousands of years (Tang et al., 2008). During China fighting against the SARS-CoV-2, more than 85% of COVID-19 patients received TCM treatment and clinical results show that TCM has a significant therapeutic on alleviating the symptoms of mild patients (Yang et al., 2020; An et al., 2021; Huang, 2021; Li et al., 2021). Among these TCMs, the “three formulas and three medicines” namely Qingfei Paidu decoction, Huashi Baidu formula, Xuanfei Baidu formula, Jinhua Qinggan granules, Lianhua Qingwen capsule, Xuebijing injection with obvious curative effects have been screened out and strongly recommended for the treatment of COVID-19 (Chen et al., 2020; Guo et al., 2020; Liu et al., 2020; Tao et al., 2020; Zheng et al., 2020; Niu et al., 2021). Moreover, “three formulas and three medicines” were listed as recommended medicine for the suspected SARS-CoV-2 patients during the medical observation period in the novel coronavirus infection pneumonia diagnosis and treatment standards (Trial fourth edition, Fifth Edition, Sixth Edition, Seventh Edition) (Zhao et al., 2020). However, some Chinese herbal products have been contaminated with toxic compounds, heavy metals, pesticides, and microorganisms and may have serious side effects. In addition, their antiviral mechanisms and specific active monomers are still unclear, more evidence are needed to support the anti-SARS-CoV-2 effect of these TCMs. Therefore, the identification of the active ingredients of these TCMs is of extraordinary significance for the clinical treatment of COVID-19 and the research of drug mechanism.

To deal with sudden and emerging infectious diseases, drug repurposing is the fastest and most effective strategy to solve the problem. Here, based on the pangolin coronavirus GX_P2V drug screening alternative model, we screened out monomer compounds with antiviral activity from the traditional Chinese medicine “three drugs and three prescriptions”. These monomer compounds also have good antiviral effects on SARS-CoV-2, which proves the effectiveness of the GX_P2V drug screening model we established.

## Materials and Methods

### Cells and Virus

The African green monkey kidney cell line Vero E6 was obtained from American Type Culture Collection (ATCC, Manassas, VA, USA) and was maintained in Dulbecco’s modified Eagle’s medium (DMEM, HyClone, Utah, USA) containing 10% fetal bovine serum (FBS) and 1% antibiotic-antimycotic (Gibco, NY, USA). The SARS-CoV-2-related coronaviruses strain GX_P2V (Pangolin coronavirus isolate PCoV_GX-P2V, accession No. MT072864.1) was isolated using Vero E6 cells from smuggled dead *Manis javanica* intercepted in 2017. With the ethics approval (wild animal treatment regulation No. [2011] 85), the Guangxi Zhuang Autonomous Region Terrestrial Wildlife Medical-aid and Monitoring Epidemic Diseases Research Center rescued and treated the animals. The samples were collected following the procedure guideline (Pangolins Rescue Procedure, November 2016). Severe acute respiratory syndrome coronavirus 2 isolate WIV04 (accession No. MN996528.1) was isolated from the bronchoalveolar lavage fluid of COVID-19 patients in Wuhan Jinyintan Hospital. The samples were collected by Jinyintan hospital (Wuhan, China) with the consent of all patients and approved by the ethics committee of the designated hospital for emerging infectious diseases. All cells and virus were cultured in a 37°C, 5% CO_2_ incubator. The infection experiments of GX_P2V and WIV04 were performed in the Level 2 Biosafety (BLS-2) laboratory of the Biosafety Technology Research Center of Beijing University of Chemical Technology and the Level 3 Biosafety (BLS-3) laboratory of Wuhan Institute of Virology, respectively.

### Preliminary Drug Screening Using GX_P2V *In Vitro*

The anti-COVID-19 traditional Chinese medicine compound library (TargetMol, Catalog No. L6720, Shanghai, China) containing 389 active monomers extracted from Chinese medicine prescriptions was purchased from Topscience (Shanghai, China). Vero E6 cells were pre-seeded in 96-well plates at a density of 5000 cells/well and continued to be cultured for 12 hours. After aspirating the supernatant and washing with PBS, the virus GX_P2V (MOI = 0.01) and various drugs with a final concentration of 50 μM was added. At 2 h post-infection (h.p.i.), the virus–drugs mixture was removed and fresh culture medium containing 10 μM drugs were added. At 48 h.p.i., cell images were taken to observe cytopathic effect (CPE), then the supernatant was discarded, and cells were collected, and the viral yield was quantified by qRT-PCR.

### Half Maximal Effective Concentration and Half-Cytotoxic Concentration Assay

In order to evaluate the antiviral effects of several drugs obtained in the preliminary screening, different doses of drugs were used for treatment while GX_P2V (MOI = 0.01) infected cells. At 2 h.p.i., the virus–drugs mixture was removed and fresh culture medium containing different doses of drugs were added. At 72 h.p.i., the supernatant was discarded and then the viral yield in the cell was quantified by qRT-PCR. The cytotoxicity of these drugs to Vero E6 cells was measured by CellTiter-Blue Cell Viability Assay (Promega, Madison, WI, USA) using Synergy H1 Hybrid Multi-Mode Microplate Reader (BioTek, Winooski, Vermont, USA) according to manufacturer’s instructions.

### Time-of-Addition (TOA) Assay and Western Blot

Elucidating the mechanism of action of drugs is an important step to facilitate further development. To this end, we performed a time-of-addition assay for these 6 drugs. Vero E6 cells were treatd with a single dose of drugs at different stages of viral infection. For “Full-time” treatment, the cells were treated with drugs immediately after infection with the virus and incubated in the 37°C incubator with 5% CO_2_ for 2 hours to allow the virus to adsorb. Afterwards, the virus–drug mixture was removed, and the cells were cultured with drug-containing medium until the end of the experiment. For “Entry” treatment, the drugs were added to the cells immediately after infection with the virus and at 2 h.p.i., the virus–drug mixture was replaced with fresh culture medium and maintained till the end of the experiment. For “Post-entry” experiment, drugs were added at 2 h.p.i., and maintained until the end of the experiment. For all the experimental groups, cells were infected with GX_P2V at an MOI of 0.01, and virus yield in the infected cells was quantified by qRT-PCR and NP expression in infected cells was analyzed by western blot at 72 h.p.i.

### Anti-SARS-CoV-2 Activities *In Vitro*

To verify the drugs screened through the GX_P2V drug screening alternative model, we repeatedly tested the antiviral activity of the above drugs on SARS-CoV-2. In brief, Vero E6 cells were pre-seeded in 48-well plates at a density of 5×10^4^ cells/well and continued to be cultured overnight. Two hours before infection, the culture supernatant of Vero E6 cells was discarded and replaced with a DMEM medium containing different concentration gradient drugs. Vero E6 cells were continued to be cultured for 2 hours, which was conducive to the early entry of the drugs into the cells. Vero E6 cells were infected with WIV04 at a MOI=0.01 for 1 h. After discarding the supernatant, the cells were washed twice with DMEM, and the medium was changed to a DMEM medium (10%FBS) containing the corresponding concentration of the drug. After 24 h.p.i, the cell supernatant was collected. According to the manufacturer’s instructions, the viral RNA was extract using Magnetic Bead Virus RNA Extraction Kits (Shanghai Fine Gene Biotech, FG438). The viral yield in the cell supernatant was then quantified by qRT-PCR using HiSxript II One step qRT-PCR SYBRGreen Kit (Vazyme, Nanjing, China).

### RNA Extraction, Reverse Transcription and Real-Time Fluorescence Quantitative PCR (q-PCR)

To assess viral loads, we extracted total RNA from the cells and performed q-PCR after reverse transcription. In brief, total RNA was extracted using an AxyPrep™ Multisource Total RNA Miniprep Kit (Axygen, Suzhou, China) in accordance with the manufacturer’s instructions. Reverse transcription was performed by a Hifair II 1st Strand cDNA Synthesis Kit with gDNA digester (Yeasen Biotech, Shanghai, China), followed by using Hieff qPCR SYBRGreen Master Mix (Yeasen Biotech, Shanghai, China) in QuantStudio 1 Real-Time PCR detection system (Applied Biosystems, CA, USA) for quantification. The primer sequences were shown in **Supplementary Table S3**. The GX_P2V S gene and GAPDH gene were cloned into the T vector to construct a recombinant plasmid to generate the plasmid standard. In each run, serial dilutions of the standard were used to calculate copy numbers in each sample.

### Statistical Analysis

Statistical analyses were analyzed using GraphPad Prism 8 software (GraphPad Software Inc., San Diego, CA, USA). Values are shown as mean of triplicates.

## Results and Discussion

### Pangolin Coronavirus GX_P2V Is an Excellent Alternative Model for SARS-CoV-2 Drug Screening

**Figure 1:**
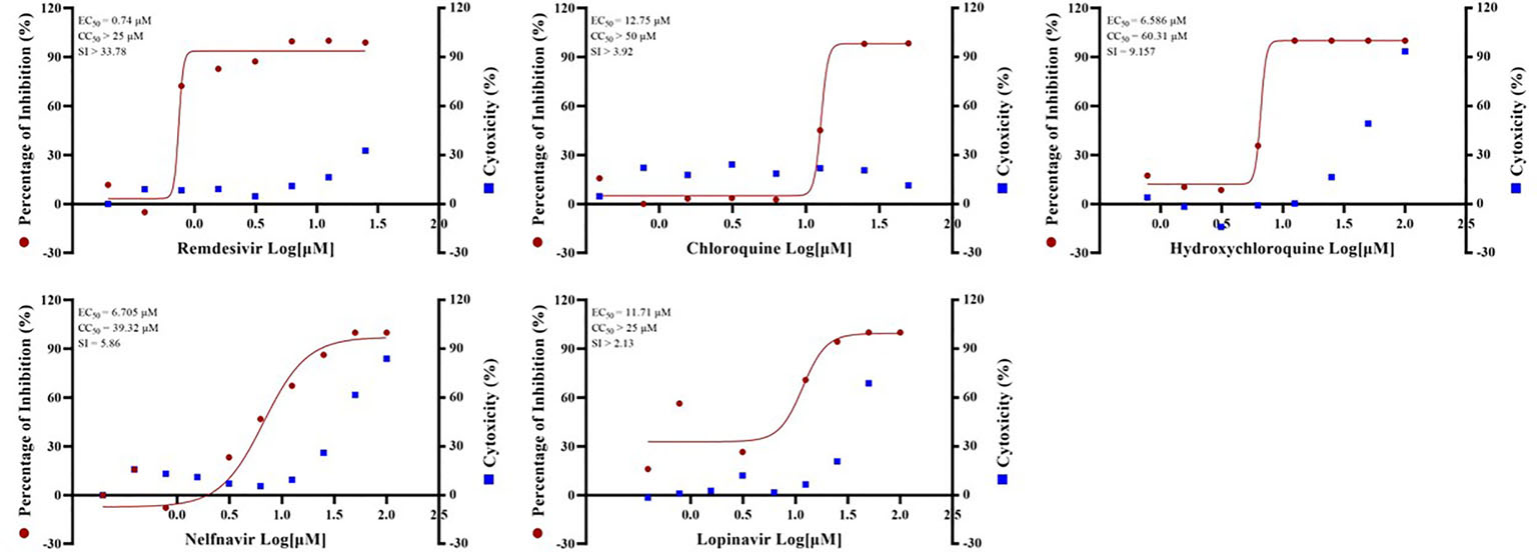
EC_50_ and CC_50_ of remdesivir, chloroquine, hydroxychloroquine, nelfinavir and lopinavir for GX_P2V. Vero E6 cells were infected with GX_P2V at an MOI of 0.01. Using different doses of remdesivir, chloroquine, hydroxychloroquine, nelfinavir and lopinavir for treatment. After 72 hours, cells were collected and total RNA was extracted, and virus yield was quantified by qRT-PCR. Cytotoxicity of these drugs to Vero E6 cells was measured by CellTiter-Blue^®^ Cell Viability Assay (Promega). The left and right Y axes of the graph represent the average percentage of inhibition of virus yield and drug cytotoxicity, respectively. The experiments were performed in triplicates.

Standard assays were implemented to measure the effects of the compounds on cytotoxicity and viral infection as previously reported (Fan et al., 2020). The previously reported anti-SARS-CoV-2 drugs including remdesivir (EC_50_ = 0.74 µM, CC_50_ > 25 µM, SI > 33.78), chloroquine (EC_50_ = 12.75 µM, CC_50_ > 50 µM, SI > 3.92), hydroxychloroquine (EC_50_ = 6.586 µM, CC_50_ = 60.31 µM, SI = 9.157), nefinavir (EC_50_ = 6.705 µM, CC_50_ = 39.32 µM, SI = 5.86) and lopinavir (EC_50_ = 11.71 µM, CC_50_ > 25 µM, SI > 2.13) **(** **Figure 1** **)** were also found to potently inhibit viral infection in our GX_P2V model. In addition, their anti-SARS-CoV-2 effect were also confirmed by other groups (Wang M et al., 2020; Weston et al., 2020; Jan et al., 2021). Therefore, these results together strongly demonstrated that GX_P2V could be used as a good alternative model for SARS-CoV-2 drug screening.

### Six Monomer Compounds Extracted From TCM Have Good Anti-GX_P2V Effect

**Figure 2:**
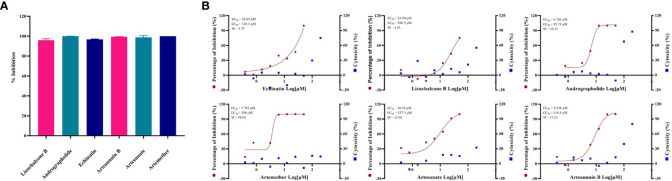
**(A)** Evaluation of anti-GX_P2V effect of monomer compounds extracted from traditional Chinese medicine. Vero E6 cells were infected with GX_P2V at an MOI of 0.01. Treating with 389 different kinds of monomer compounds in the “three formulas and three medicines”, the final concentration of each drug was 50 μM, and after 48 h, total RNA was extracted, and qRT-PCR was performed. The Y axis of the graph represents the average inhibition rate of the virus. The X-axis of the graph represents six drugs that have excellent anti-coronavirus activity. The experiments were done in triplicates. **(B)** EC_50_ and CC_50_ of six monomer compounds in “Three Medicines and Three Prescriptions”. Virus infection and drug treatment were performed as mentioned above. Among the drugs obtained in the preliminary screening, 6 drugs have excellent antiviral activity and low cytotoxicity, and have the potential to become specific drugs for the treatment of COVID-19. The experiments were done in triplicates.

By screening the antiviral effects of 389 monomer compounds in the “three formulas and three medicines” (TargetMol, L6720, **Supplementary Table S1**) at the final concentration of 50 μM, six of the monomer compounds have been found to reduce the GX_P2V infection remarkably **(** **Figure 2A**, **Supplementary Figure S1** and **Supplementary Table S2** **)**. Among these six drugs, high concentrations of two anticancer drugs including echinatin (Oh et al., 2020) (EC_50_ = 20.89 µM, CC_50_ = 120.1 µM, SI = 5.75), and licochalcone B (Song et al., 2020) (EC_50_ = 24.90 µM, CC_50_ = 106.5 µM, SI = 4.28) were required to inhibit the viral infection **(** **Figure 2B** **)**. Notably, andrographolide (Enmozhi et al., 2020) (EC_50_ = 6.786 µM, CC_50_ = 95.73 µM, SI = 14.11) were also found to block the virus infection **(** **Figure 2B** **)**. It is interesting and refreshing to find that three derivatives of the well-known antimalarial drug artemisinin (Tu, 2016), namely, artemether (EC_50_ = 3.701 µM, CC_50_ > 200 µM, SI > 54.04), artesunate (EC_50_ = 10.10 µM, CC_50_ = 127.3 µM, SI = 12.60) and arteannuin B (EC_50_ = 8.838 µM, CC_50_ = 116.9 µM, SI = 13.23) can effectively reduce the viral infection **(** **Figure 2B** **)**, strikingly, all of these three drugs can be extracted from Artemisia annua, which exists in Reduning injection, Toujiequwen granules and Jinhua Qinggan granules **(** **Supplementary Table S2** **)**. However, considering that our study measured the inhibitory effects of these six monomer compounds on the virus at a low MOI, this may overestimate the antiviral effects of these drugs because the interferon response may be involved. The inhibitory effect of the drugs on the GX_P2V and SARS-CoV-2 infection under the high MOI (preferentially at 1-5) is expected in the future.

### All These Drugs Functioned at the Post-Entry Stage and Strongly Inhibit NP Protein Expression

**Figure 3:**
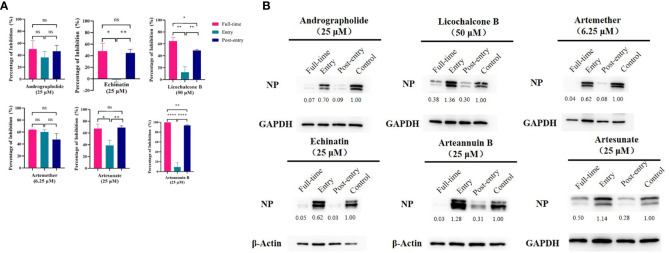
**(A)** Time-of-addition assay and western blot of six monomer compounds in “Three Medicines and Three Prescriptions”. For “Full-time” treatment, the cells were treated with drugs immediately after infection with GX_P2V and incubated in the 37°C incubator with 5% CO_2_ for 2 hours to allow the virus to adsorb. Afterwards, the virus–drug mixture was removed, and the cells were cultured with drug-containing medium until the end of the experiment. For “Entry” treatment, the drugs were added to the cells immediately after infection with the virus and at 2 h.p.i., the virus–drug mixture was replaced with fresh culture medium and maintained till the end of the experiment. For “Post-entry” experiment, drugs were added at 2 h.p.i., and maintained until the end of the experiment. For all the experimental groups, cells were infected with GX_P2V at an MOI of 0.01, and virus yield in the infected cells were quantified by qRT-PCR and **(B)** NP expression in infected cells was analyzed by western blot at 72 h.p.i. The experiments were done in triplicates. *p < 0.1, **p < 0.01, ****p < 0.0001, ns, not significant.

The time-of-addition assay **(** **Figure 3A** **)** and western blot **(** **Figure 3B** **)** showed that, among the six monomer compounds screened in the previous step, except for artemether, the remaining five drugs all functioned at the post-entry stage. And artemether functioned on both entry and post-entry stages. Among them, when artemether was added at 6.25 µM, the expression of viral NP protein was almost completely inhibited. 25 µM arteannuin B and 25 µM andrographolide also achieved the same inhibitory effect and most viral NP proteins can be inhibited by 25 µM echinatin, 25 µM artesunate, and 50 µM licochalcone B. As previous research (Shi et al., 2020; Sukardiman et al., 2020), andrographolide and its derivative inhibit SARS-CoV-2 by covalently linkage with main proteases. However, the targets of several other drugs are not yet clear, and further study of the mechanism of action of these drugs is of great significance to their promotion and wide-scale use.

Artemisinin possesses antiviral effects against a variety of viruses. A recent study evaluated the antiviral activity of artemisinin against flaviviruses such as JEV, DENV and ZIKV *in vitro* and *in vivo* and found that the antiviral effect induced by artemisinin was associated with enhanced host type I interferon response (Wang X et al., 2020). In addition, the expression of interferon-stimulated genes induced by artemisinin will be inhibited by the blocking of interferon signaling. Artesunate may play a role at the level of cell signaling and might downmodulate regulatory processes, such as activating NF-κB or Sp1 pathways, thereby interfering with HCMV replication (Efferth et al., 2008; Schreiber et al., 2009). Andrographolide has antiviral activity against a number of positive-sense RNA viruses transmitted by mosquitoes, including DENV, CHIKV and ZIKV. Andrographolide may exerts anti-dengue virus effect by inducing HO-1 signal pathway (Tseng et al., 2016). And GRP78 and unfolded protein response play an important role in mediating the anti-dengue virus activity of andrographolide (Paemanee et al., 2019). In a murine model of inflammation caused by influenza A virus, andrographolide can inhibit NF-κB and JAK-STAT signaling pathways (Ding et al., 2017). The antiviral action mechanism of traditional medicine compounds in the above research will have a positive reference significance for further research on their anti-SARS-CoV-2 mechanism.

### Artesunate, Arteannuin B, Echinatin, Licochalcone B and Andrographolide Effectively Inhibit SARS-CoV-2

**Figure 4:**
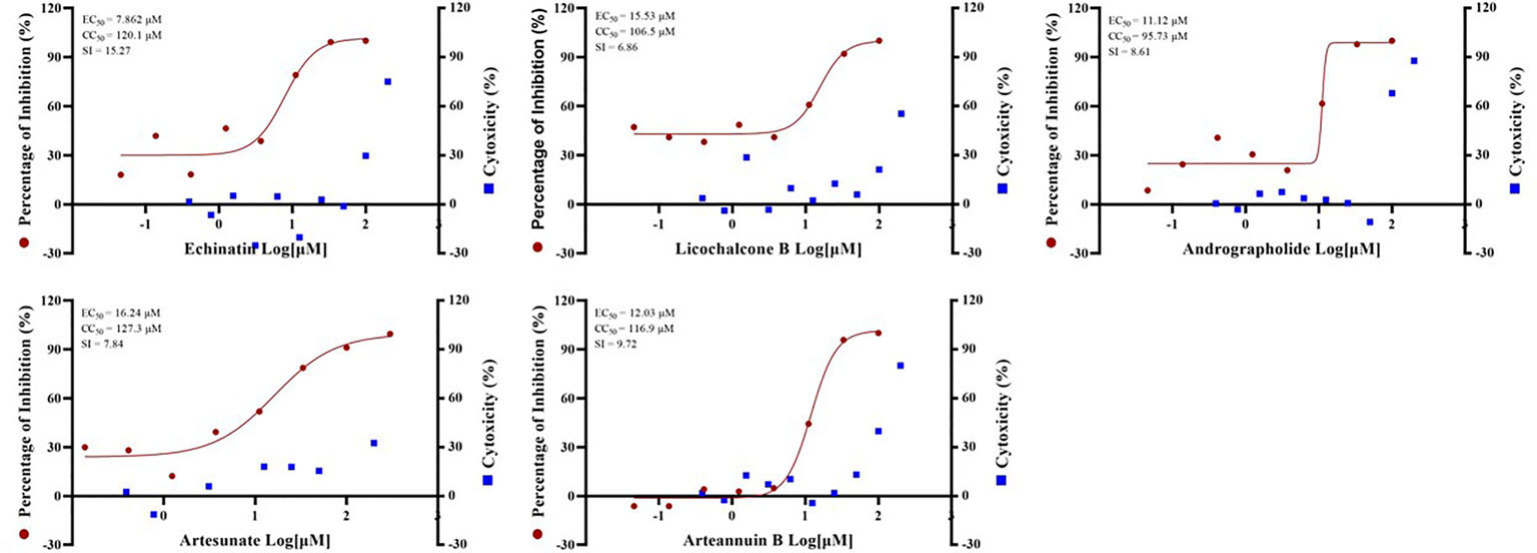
The EC_50_ and Time-of-addition assay of the five monomer compounds in the “Three Medicines and Three Prescriptions” against SARS-CoV-2. Vero E6 cells were infected with SARS-CoV-2 at an MOI = 0.01 in the treatment of different doses of the indicated antivirals for 24h. The viral yield in the cell supernatant was then quantified by qRT-PCR. The Y axes of the graph represent the average percentage of inhibition of virus production. The experiments were done in triplicates.

In addition, SARS-CoV-2 (severe acute respiratory syndrome coronavirus 2 isolate WIV04, accession No. MN996528.1) was used to verify the antiviral effects of certain drugs. And artesunate (EC_50_ = 16.24 µM, CC_50_ = 127.3 µM, SI = 7.84), arteannuin B (EC_50_ = 12.03 µM, CC_50_ = 116.9 µM, SI = 9.72), echinatin (EC_50_ = 7.862 µM, CC_50_ = 120.1 µM, SI = 15.27), licochalcone B (EC_50_ = 15.53 µM, CC_50_ = 106.5 µM, SI = 6.86) and andrographolide (EC_50_ = 11.12 µM, CC_50_ = 95.73 µM, SI = 8.61) all showed excellent anti-SARS-CoV-2 virus activity **(** **Figure 4** **)**. The anti-SARS-CoV-2 effect of these drugs is basically the same as or even better than that of GX_P2V, indicating that pangolin coronavirus GX_P2V is an excellent preliminary screening model for anti-SARS-CoV-2 drugs, which can reduce the burden on the the Level 3 Biosafety (BLS-3) laboratory.

Previous studies have found that artemisinin is a potential broad-spectrum antiviral drug. Based on this, a number of experiments have studied the antiviral effect of artemisinin on SARS-CoV-2. Cao et al. (Cao et al., 2020) evaluated the anti-SARS-CoV-2 activities of nine artemisinin-related compounds *in vitro*, and also found that artesunate and arteannuin B can effectively inhibit SARS-CoV-2, with EC_50_ of 12.98 ± 5.30 μM and 10.28 ± 1.12 μM, respectively, which are similar to the results of this study. Gendrot et al. studied antimalarial artemisinin-based combination therapies and found that mefloquine-artesunate exerts the highest antiviral activity *in vitro*, with an inhibition rate of 72.1 ± 18.3% (Gendrot et al., 2020). Nair et al. also found that artemisia annua L. extracts can inhibit the replication of SARS-CoV-2 *in vitro* and can also inhibit the variants of the United Kingdom and South Africa, B1.1.7 and B1.351 (Nair et al., 2021). Utilizing *in silico*-based techniques of molecular docking and MD simulations, Dey et al. found that artemether may be a potential SARS-CoV-2 envelope (E) protein ion channel inhibitor, revealing its anti-SARS-CoV- 2 possible targets (Dey et al., 2020).

Overall, our findings strongly suggest that artemether, artesunate, arteannuin B, echinatin, licochalcone B and andrographolide in “three formulas and three medicines” are the potential active ingredients for the inhibition of SARS-CoV-2 *in vitro*. Significantly, our study paves the way to clarify the mechanisms of traditional Chinese medicine against SARS-CoV-2.

## Data Availability Statement

The original contributions presented in the study are included in the article/**Supplementary Material**. Further inquiries can be directed to the corresponding authors.

## Ethics Statement

The studies involving human participants were reviewed and approved by the ethics committee of the Jinyintan hospital for emerging infectious diseases. The patients/participants provided their written informed consent to participate in this study. The animal study was reviewed and approved by Guangxi Zhuang Autonomous Region Terrestrial Wildlife Medical-aid and Monitoring Epidemic Diseases Research Center.

## Author Contributions

HF, XY, LS, and YT designed the research. YH, ML, HQ, and HL performed the experiments. YH, ML, XA, and ZS analyzed the data. HF and YH wrote and revised the manuscript. All authors contributed to the article and approved the submitted version.

## Funding

This research was supported by Key Project of Beijing University of Chemical Technology (No. XK1803-06), National Key Research and Development Program of China (NO. 2018YFA0903000, 2020YFC2005405, 2020YFA0712100, 2020YFC0840805, 19SWAQ06), Funds for First-class Discipline Construction (No. XK1805), Inner Mongolia Key Research and Development Program (NO. 2019ZD006), National Natural Science Foundation of China (NO. 81672001, 81621005), NSFC-MFST project (China-Mongolia) (No. 31961143024), Fundamental Research Funds for Central Universities (No. BUCTRC201917, BUCTZY2022).

## Conflict of Interest

The authors declare that the research was conducted in the absence of any commercial or financial relationships that could be construed as a potential conflict of interest.

## Publisher’s Note

All claims expressed in this article are solely those of the authors and do not necessarily represent those of their affiliated organizations, or those of the publisher, the editors and the reviewers. Any product that may be evaluated in this article, or claim that may be made by its manufacturer, is not guaranteed or endorsed by the publisher.
