# Supplementary material for: Artemether, Artesunate, Arteannuin B, Echinatin, Licochalcone B and Andrographolide Effectively Inhibit SARS-CoV-2 and Related Viruses In Vitro
Source: Front Cell Infect Microbiol. 2021 Aug 30;11:680127. doi: 10.3389/fcimb.2021.680127 (PMC8435859; doi:10.3389/fcimb.2021.680127)
Supplement: Supplementary file 1 [file DataSheet_1.docx]

Supplementary Material

# Supplementary Figures and Tables

## Supplementary Figures


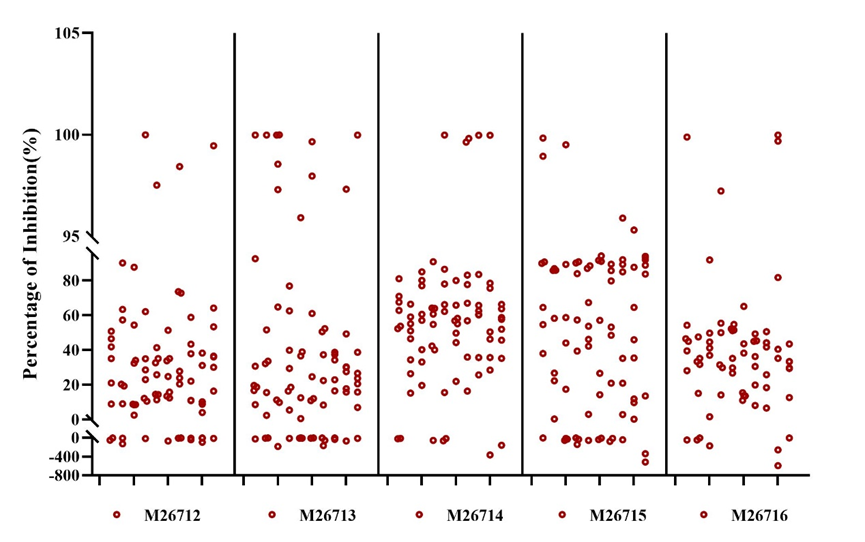


**Supplementary Figure S1.** **Anti-GX-P2V effect of 389 monomer compounds in “Three formulas and Three medicines”.** The x-axis is divided into five parts, and each part represents the 96-well plate contained in the drug library. Below the x-axis is the number of each well, from M26712 to M26716. Each hollow circle represents a medicine. The Y-axis of the graph represents the average inhibition rate of the virus.

## Supplementary Tables

**Supplementary Table S1. Information about the anti-COVID-19 Traditional Chinese Medicine Compound Library.**

**Supplementary Table S2. Sources of six monomer compounds and corresponding prescriptions.**

| **Name** | **Source** | **Source of prescription** |
| --- | --- | --- |
| Andrographolide | Andrographis paniculata | Xiyanping Injection |
| Echinatin | Glycyrrhiza uralensis | Lianhua Qingwen capsule and Xiaochaihu Decoction |
| Licochalcone B | Glycyrrhiza uralensis | Lianhua Qingwen capsule and Xiaochaihu Decoction |
| Artemether | Artemisia carvifolia | Reduning injection, toujiequwen granules and Jinhua Qinggan granules |
| Artesunate | Artemisia carvifolia | Reduning injection, toujiequwen granules and Jinhua Qinggan granules |
| Arteannuin B | Artemisia carvifolia | Reduning injection, toujiequwen granules and Jinhua Qinggan granules |

**Supplementary Table S3. Primers used in this study.**

| **Primers** | **Sequence (5’-3’)** |
| --- | --- |
| GX-P2V_Forward primer | GGTGATTGCCTTGGTGATATTG |
| GX-P2V_Reverse primer | GCAAGTAGTGCAGAAGTGTATTG |
| SARS-CoV-2_Forward primer | CAATGGTTTAACAGGCACAGG |
| SARS-CoV-2_Reverse primer | CTCAAGTGTCTGTGGATCACG |
| GAPDH_Forward primer | AGCCTCAAGATCATCAGCAATG |
| GAPDH_Reverse primer | ATGGACTGTGGTCATGAGTCCTT |
